# The University of Texas Southwestern Glioma Dataset - MRI, Molecular Markers and Segmentations

**DOI:** 10.1038/s41597-026-07274-4

**Published:** 2026-04-22

**Authors:** Divya D. Reddy, Niloufar Saadat, James M. Holcomb, Benjamin C. Wagner, Nghi C. Truong, Jason Bowerman, Kimmo J. Hatanpaa, Toral R. Patel, Marco C. Pinho, Fang Yu, Kuan Zhang, Sadeem Lodhi, Ananth J. Madhuranthakam, Chandan Ganesh Bangalore Yogananda, Joseph A. Maldjian

**Affiliations:** 1 Advanced Neuroscience Imaging Research lab, Department of Radiology, University of Texas Southwestern Medical Center, Dallas, Texas USA; 2 Department of Pathology, University of Texas Southwestern Medical Center, Dallas, Texas USA; 3 Department of Physiology and Biomedical Engineering, Mayo Clinic, Rochester, Minnesota, USA

**Keywords:** CNS cancer, Cancer imaging, Diagnostic markers, Cancer genetics, Tumour biomarkers

## Abstract

Gliomas are the most common type of primary brain tumors. Their management options and outcomes depend significantly on the underlying molecular-marker profile. Traditionally, molecular markers are determined through pathological testing on a tissue specimen acquired through biopsy. Several Magnetic Resonance Imaging (MRI) based Deep Learning (DL) methods offer a promising, non-invasive approach to predict these markers. However, they often require high-quality, well-annotated datasets. To support this need, we present a well-curated brain tumor dataset developed at The University of Texas Southwestern (UTSW) Medical Center. This dataset includes multi-contrast-MRI, demographics, molecular-markers, and multi-label tumor segmentations for 625 patients treated at UTSW between 2006 and 2023. Each patient record contains four MRI contrasts: pre-contrast-T1w, post-contrast-T1w, T2w, and T2-weighted fluid-attenuated inversion recovery (T2w-FLAIR) images. The dataset also provides comprehensive genetic information, including IDH mutation-status, 1p19q co-deletion, MGMT promoter methylation, tumor-type, and tumor-grade. This dataset offers a valuable resource for exploring the relationship between MRI characteristics and tumor genetics. It also serves as a robust benchmark for developing and validating DL models for various downstream tasks.

## Background & Summary

Gliomas are the most common primary brain tumors^1^. They can present significant challenges in clinical diagnosis and treatment^2^. However, genetic testing and molecular profiling have transformed diagnosis and treatment approaches^3,4^. The 2021 WHO glioma re-classification introduced critical molecular markers, such as MGMT methylation, IDH mutation, and 1p19q co-deletion, into diagnostic protocols^5–7^. These molecular markers combined with traditional histological assessments enable more effective treatment strategies^8^.

Recent advances in deep learning (DL) and medical imaging have offered promising non-invasive alternatives for profiling molecular markers^9–17^. Such DL models are effective in recognizing complex imaging patterns across large datasets, aiding in prognosis and treatment planning^18^. However, the performance of these models depends on the quality and completeness of the training/testing data. Factors such as image quality, accurate tumor labeling, and comprehensive genetic information are essential for achieving reliable results^19–21^. The importance of well-curated datasets is highlighted by efforts including the BraTS21 challenge^22^, UCSF^23^, UPenn^24^, Erasmus^25^, and TCIA^26^ repositories, which have provided a large collection of brain tumor MRI with expert labels. These resources have significantly advanced the field by supporting the development and validation of DL models for several tasks including molecular profiling.

Building such high-quality datasets is a complex task that requires careful preparation and validation. Ensuring accuracy and consistency is essential for models to perform reliably across diverse clinical settings. We have curated a brain tumor dataset that integrates multi-contrast MRI, genetic information, and other clinical variables from patients with primary brain tumors treated at UTSW Medical Center between 2006 and 2023. The UTSW Glioma dataset specifically includes a) pre-operative MRI scans (pre-contrast-T1w, post-contrast-T1w, T2w, and T2w-FLAIR), b) clinical and demographic information such as age, sex, race, ethnicity, tumor type and tumor grade and c) molecular profiles including IDH mutation, 1p19q co-deletion, and MGMT promoter methylation status and d) multi-label tumor segmentations. Through this dataset, we aim to support researchers in advancing AI-driven approaches for the diagnosis and treatment of brain gliomas.

## Methods

This section outlines the procedures used to curate the UTSW Glioma dataset, including patient selection; acquisition of demographic, molecular, and imaging data; image quality assessment; computational pre-processing; data curation and tumor segmentation.

### Patient selection

Subjects were shortlisted based on a neuropathologist-led review of electronic health records (EHR) for patients diagnosed with glioma at UTSW between 2006 and 2023. A total of 1061 subjects were identified in this process. These subjects were then screened to meet the following inclusion criteria: (i) age > 18 years, (ii) no prior history of tumor resection, and (iii) availability of multi-contrast preoperative MRI (pre-contrast T1-weighted images (T1w), postcontrast T1-weighted images (T1w + C), T2-weighted images (T2w), and T2-weighted fluid attenuated inversion recovery (T2w-FLAIR)) (iv) testing of one or more molecular markers (v) MRI scans acquired within one year of the histopathological analysis and (vi) images that satisfied quality assessment standards. The final dataset included 625 subjects which met the inclusion criteria. Institutional Review Board (IRB) approval was obtained for the use of patient data, and informed consent was waived due to the retrospective nature of the study (STU-2020-1184). The shared data are skull-stripped and therefore fully anonymized and non-identifiable.

### Data acquisition

**Fig. 1:**
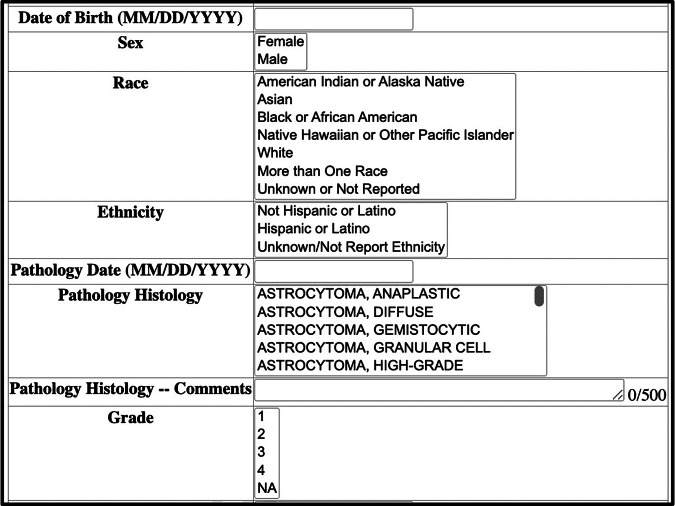
Internal XNAT Reporting System. This figure shows an XNAT-based internal reporting system designed to extract and standardize data from electronic health record (EHR) systems. It captures key demographic, histopathological and genetic information including age, sex, race, ethnicity, pathology, surgery dates and molecular marker status.

Clinical and molecular marker information were extracted from the EPIC EHR system (Epic Systems Corporation, Verona, WI). An internal reporting system was developed using the XNAT^27^ imaging informatics platform to manage and organize the dataset (Fig. 1). This included essential demographic details such as age, sex, race, ethnicity, pathology, surgery dates, and molecular marker status (Table 1 and UTSW metadata file).

**Table 1:** Demographics of the UTSW dataset.

Demographics	Value	Number	Percentage
Gender	Female	254	40.64%
	Male	371	59.36%
Age	18–29	48	7.68%
	30–49	158	25.28%
	50–69	297	47.52%
	70 +	122	19.52%
Race	White	538	86.08%
	American Indian/ Alaskan Native	2	0.32%
	Asian	5	0.80%
	Black or African American	15	2.40%
	Unknown/ Other	65	10.40%
Ethnicity	Non-Hispanic/ Latino	554	88.64%
	Hispanic/ Latino	12	1.92%
	Unknown/ Other	59	9.44%

The study population comprised of 371 males (59.36%) and 254 females (40.64%). Age was calculated with reference to the time of scan. The range of the included population is 18–85 years of age, with the mean age being 55 years. 7.68% of the subjects were between 18–29 years of age, 25.28% between 30–49, 47.52% between 50–69 and 19.52% above 70 years of age.

### Molecular profiling

**Fig. 2:**
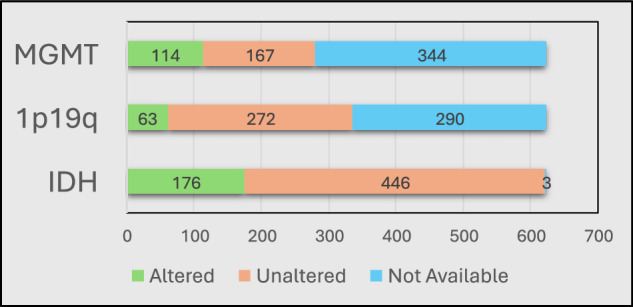
Distribution of Molecular Marker Statuses in the UTSW Dataset. The figure shows the distribution of molecular marker statuses (MGMT, 1p19q, and IDH) across the dataset. “Altered” indicates the presence of the relevant molecular marker: MGMT promoter methylation (n = 114), 1p19q co-deletion (n = 63), and IDH mutation (n = 176). “Unaltered” corresponds to unmethylated MGMT (n = 167), non-co-deleted 1p19q (n = 272), and IDH wildtype (n = 446). Cases lacking available molecular data are shown in blue.

**Fig. 3:**
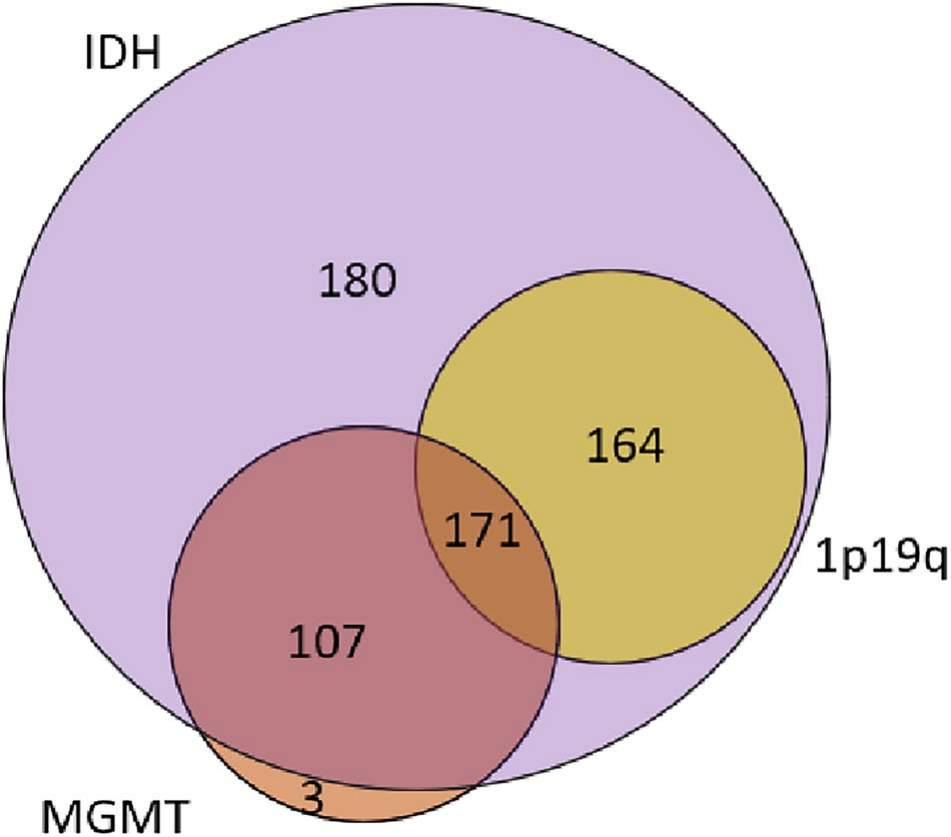
Availability and Overlap of Molecular Marker Information Across the Dataset. This figure illustrates the availability of IDH, 1p19q, and MGMT status and their overlap across subjects. IDH status was the most frequently available marker (n = 622), encompassing all cases with 1p19q information. Interestingly, three subjects had MGMT data available despite missing IDH results, contrary to the usual testing sequence. A total of 171 cases had complete molecular profiles for all three markers.

**Fig. 4:**
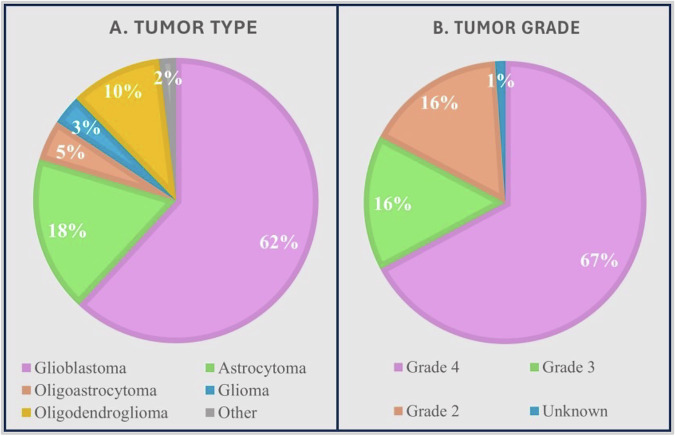
Tumor Type and Grade Distribution in the UTSW Dataset. (**A**) shows the distribution of glioma subtypes, with glioblastoma being the most common tumor type (62%), followed by astrocytoma and generic glioma. (**B**) depicts tumor grade distribution, where the majority of cases were classified as WHO grade 4, indicating a high proportion of aggressive, high-grade tumors in the cohort.

Molecular marker status was determined based on genetic analyses performed on tumor tissue collected during routine biopsy or tumor resection following the current WHO classification of CNS tumors^5,6^. The choice of testing method for assessing IDH – Next-Generation Sequencing (NGS) or Immunohistochemical (IHC) staining for IDH1-R132H – depended on the quantity and quality of available tissue. The specific testing modality used for each subject is provided in the accompanying metadata file to ensure transparency in IDH classification. IDH status was identified for 622 cases and not available for 3 cases. Mutations in IDH were found in 176 subjects (28.16%), while the rest were classified as IDH wildtype (IDHwt). MGMT (O6-methylguanine-DNA-methyltransfarase) promoter methylation was evaluated using quantitative methylation-specific polymerase chain reaction (PCR) with the cutoff set at 10%. MGMT status was available for 281 subjects: methylation was detected in 114 subjects and not detected in 167 subjects. 1p19q co-deletion status was determined using NGS or PCR-based loss of heterozygosity (LOH) analysis^28^. 63 co-deleted and 272 non-co-deleted subjects were identified. Molecular marker characterization within the patient population is summarized in Figs. 2 & 3. Tumor specimens were analyzed to determine tumor type and tumor grade according to the WHO 2021 criteria^5,6^, revealing glioblastoma as the most frequently diagnosed tumor type. This highlights the aggressive nature of gliomas in the studied population. The diagnosis of oligoastrocytoma, now considered obsolete under current WHO CNS tumor classification guidelines, was applied in earlier cases that lacked comprehensive molecular characterization. These tumors were classified based solely on histopathological features. The distribution of additional glioma subtypes is provided in Fig. 4A,B.

### Data and preprocessing

MRI scans for each subject were initially reviewed using the clinical Picture Archiving and Communication System (PACS) to identify pre-surgical timepoints. The selected scans were exported (DICOM) and uploaded to the internal XNAT server. An in-house automated pipeline was used to convert the DICOM images into NIfTI (Neuroimaging Informatics Technology Initiative) format for further computational processing. The dataset consists of MR images acquired using scanners from multiple vendors including GE, Siemens, Hitachi, Toshiba, and Philips, with magnetic field strengths ranging from 0.3 Tesla to 3 Tesla. Detailed scanner specifications are provided in the metadata file. Given the variability in imaging protocols across different machines, the FeTS platform was used for pre-processing^29^. FeTS registered the MRI scans to the SRI24 brain atlas, standardizing the resolution to 1 mm per voxel. It also performed skull-stripping to remove non-brain structures and segmented the tumor into three key regions: the enhancing tumor (ET), the non-enhancing necrotic core (NCR), and the surrounding edematous or infiltrated tissue (ED)^30,31^.

### Image quality assessment

**Fig. 5:**
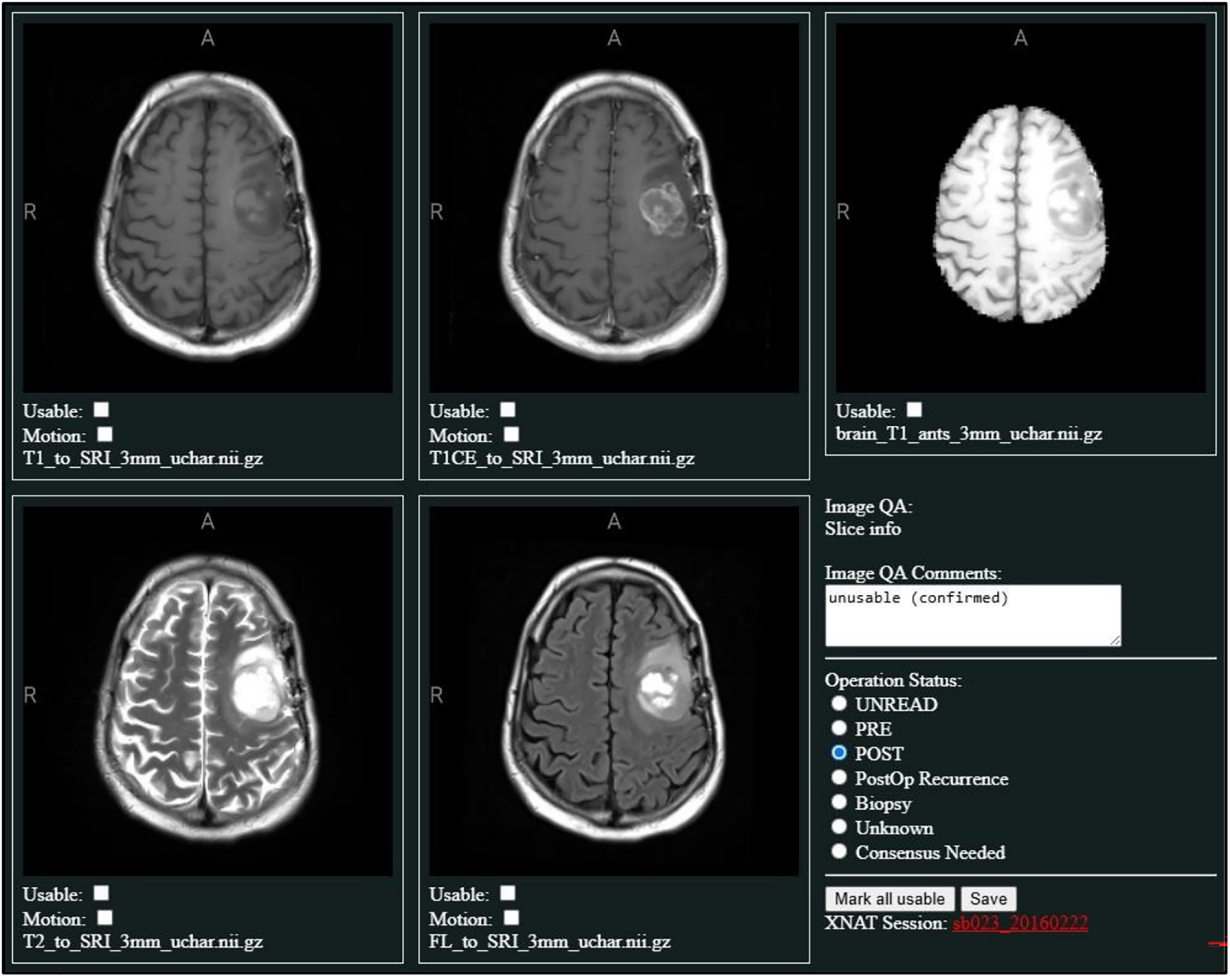
Internal XNAT Tool for MRI Quality Assessment. This figure shows an internal XNAT QA tool to screen preoperative MRI scans. The displayed case was flagged during review due to a left-sided craniotomy. Upon neuroradiologist confirmation, the case was excluded from the final dataset. QA flags like this help ensure the dataset includes only untreated, preoperative images suitable for downstream analysis. The case clearly demonstrates the surgical defect, underscoring the importance of visual inspection during data curation.

MR image quality was initially assessed by trained research staff and then confirmed by expert neuroradiologists using a custom-built internal XNAT tool (Fig. 5). The assessment focused on identifying motion/noise artifacts, evaluating skull-stripping accuracy, and distinguishing signs of prior surgery or biopsy. MRIs with poor quality due to patient motion and/or scanner artifacts were excluded. MRIs with post-surgical cavities were classified as post-operative cases, and those showing small cranial openings (e.g., burr holes) without significant tissue removal were labeled as biopsy cases. Both post-operative and biopsy cases were excluded from the dataset, resulting in a curated set of high-quality, preoperative MRIs suitable for downstream use.

### Tumor segmentation

**Fig. 6:**
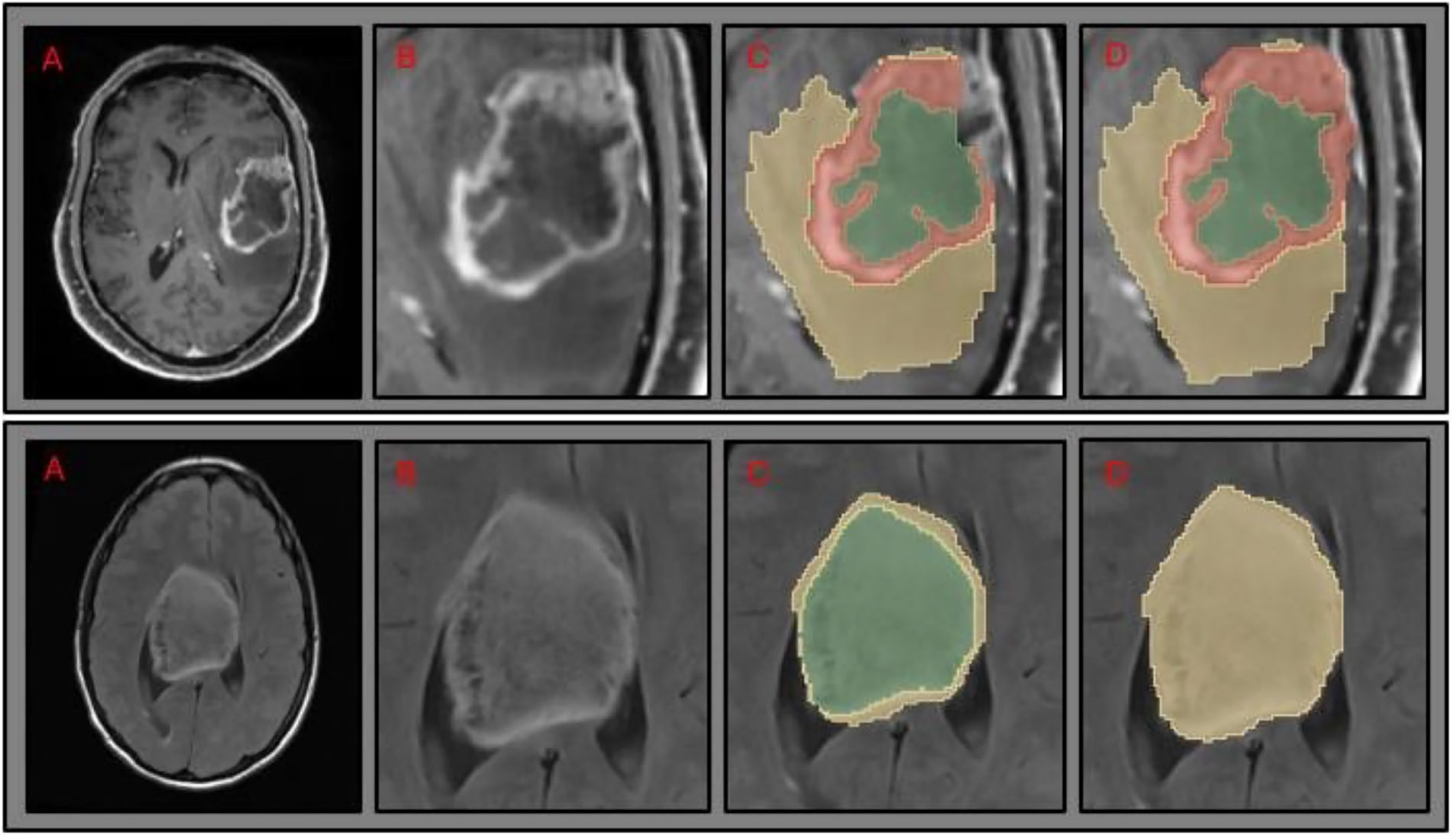
Tumor Segmentation Outputs: Automated vs. Manually Refined. This figure compares automated and manually corrected tumor segmentations for two representative cases, one with an enhancing tumor (top row) and one with a non-enhancing tumor (bottom row). (**A**) Axial slice of a tumor MRI, **(B)** Zoomed-in view of the tumor region to better visualize structural details, (**C**) Automated segmentation generated by FeTS, enhancing tumor (ET, red), necrotic core (NCR, green), and peritumoral edema (ED, yellow) and, (**D**) Manually refined segmentation after expert review. The FeTS segmentations for the non-enhancing tumor required major corrections, including removal of an incorrectly labeled necrotic core. This comparison highlights the importance of manual refinement in improving segmentation accuracy, particularly for challenging tumor presentations.

**Fig. 7:**
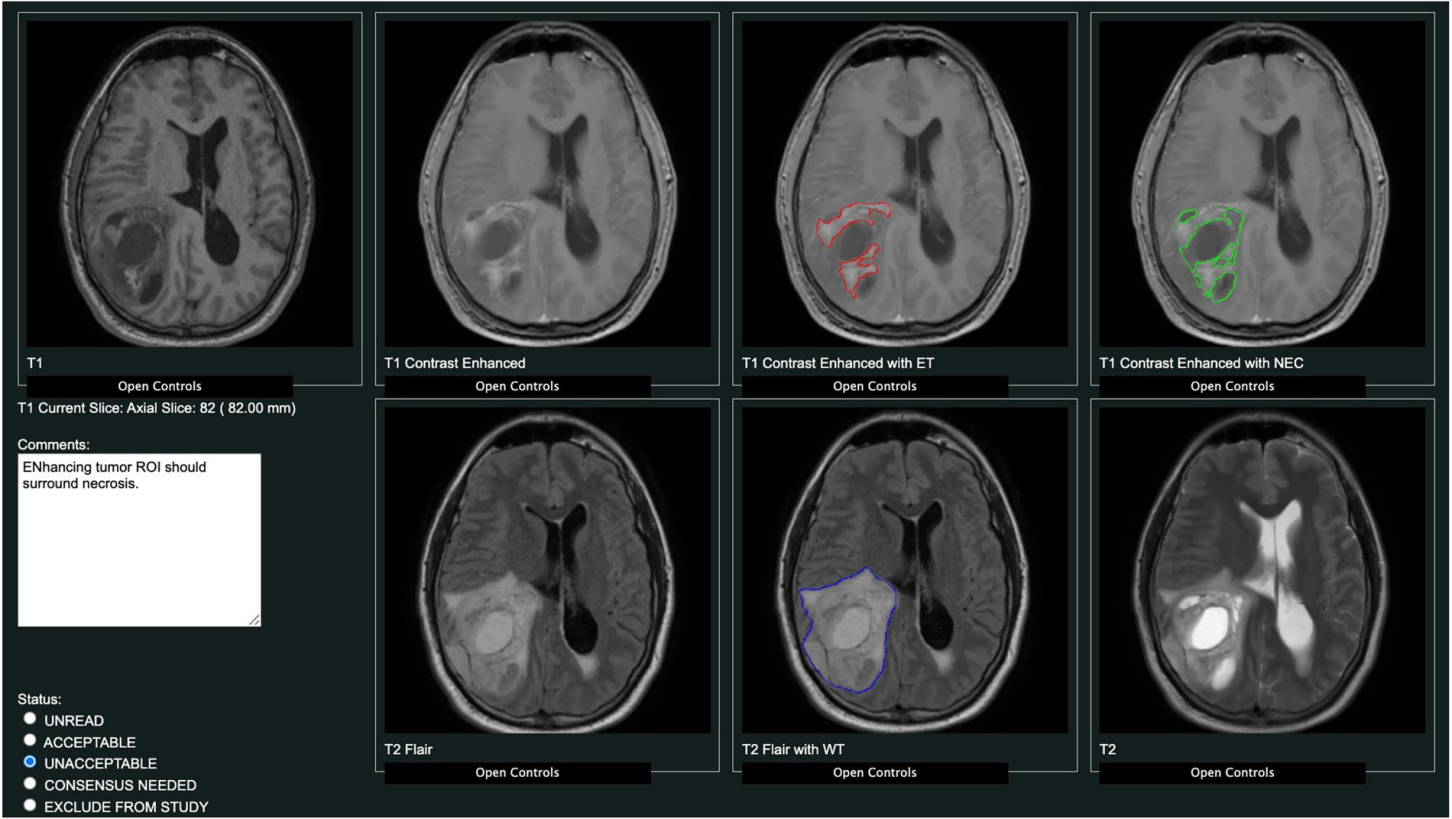
Internal XNAT Tool to review tumor segmentations. This figure shows an internal QA tool used to review and verify tumor segmentations. The displayed case illustrates a segmentation flagged as unacceptable during neuroradiologist review. The necrotic core (NCR) label was incorrectly assigned to areas not fully enclosed by the enhancing tumor (ET). Such errors prompted iterative revisions highlighting the value of expert review in ensuring segmentation accuracy.

Brain tumors were categorized into three regions: the enhancing tumor (ET), necrotic tumor core (NCR), and peritumoral edema (ED), as described in the FeTS protocol^29,31^. Initial tumor segmentations (all three regions) were generated using the FeTS platform. These segmentations were then manually reviewed and refined (3D Slicer^32^) by trained research staff to improve anatomical accuracy. During the manual editing and review, multi-contrast images (T1w, T1w + C, T2w, and T2w-FLAIR) were used to improve the distinction of complex tumor structures. Relative intensities between the pre-contrast T1-weighted and the post-contrast T1-weighted MR images were compared, to delineate the ET and NCR parts of the tumor. The hyper-intense areas were marked as ET and hypo-intense areas completely encircled by the ET regions as NCR. The edematous tumor tissues were identified based on the abnormal hyper-intense regions on T2weighted FLAIR images. An example of the segmented tumor sub-regions is shown in Fig. 6. Expert neuroradiologists subsequently evaluated the edited segmentations for (i) under or over segmentation of ROIs (ii) voxels with white matter disease classified as ED (iii) unclassified voxels (holes) within the tumor core (iv) non enhancing tumor (ED) voxels classified as NCR and (v) hemorrhage being classified as ET. Cases that did not meet the desired quality were marked as unacceptable and flagged for further revision as illustrated in Fig. 7. When necessary, segmentations underwent iterative review cycles involving both research staff and neuroradiologists to resolve discrepancies and enhance delineation precision. Some complex segmentations were flagged for consensus simultaneous review by a group of 4 neuroradiologists. A final quality check by neuroradiologists ensured that all segmentations met stringent standards before being included in the dataset. This structured, multi-stage process enabled the creation of 362 high-quality, manually refined tumor segmentations, ensuring their reliability for downstream research applications.

## Data Records

All the data described above are released as a part of the “UTSW-Glioma” dataset hosted by The Cancer Imaging Archive (TCIA), https://www.cancerimagingarchive.net/collection/utsw-glioma/, a publicly available repository^33^. The dataset comprises multi-parametric MRI scans, with skull-stripped variants, along with corresponding tumor segmentation masks, all provided in Neuroimaging Informatics Technology Initiative (NIfTI) format. Skull-stripped images were generated using two preprocessing pipelines: the Federated Tumor Segmentation (FeTS) pipeline and the Advanced Normalization Tools (ANTs) software package^34^, allowing selection of appropriate inputs for downstream analysis workflows. The accompanying metadata are provided as a tab-separated values (.tsv) file, containing patient-level information on demographics, tumor characteristics, and MRI acquisition parameters. Each row corresponds to a single patient, with columns representing variables such as anonymized patient identifier, age, sex, race, ethnicity, histopathological diagnosis, tumor grade, molecular marker status and scanner information.

## Technical Validation

### Molecular characterizations

Molecular marker information of the subjects included in the UTSW dataset obtained retrospectively from EPIC were carefully reviewed. Cases exhibiting atypical or discordant molecular profiles—such as *IDH* wildtype status accompanied by 1p19q codeletion—were re-evaluated by a board-certified neuropathologist according to the WHO 2021 criteria.

### Image quality and segmentations

All images were subjected to a structured quality control process as outlined in the Image Quality Assessment section. Cases with indeterminate operative status were initially labeled as ‘consensus needed’ and later reviewed by the full panel of 4 neuroradiologists to establish a final classification. Similarly, tumor segmentations with a high degree of uncertainty-particularly regarding the delineation of specific tumor subregions – were reevaluated by the entire curation team. Consensus was achieved through collaborative review and segmentation labels were adjusted accordingly. Final segmentations were marked as acceptable only after all neuroradiologists’ comments had been addressed to satisfaction.

## Data Availability

The UTSW-Glioma dataset is publicly available through The Cancer Imaging Archive (TCIA) at: https://www.cancerimagingarchive.net/collection/utsw-glioma/. The dataset includes MRI scans and corresponding tumor segmentations provided in Neuroimaging Informatics Technology Initiative (NIfTI) format. Associated metadata, including demographic, histopathological, and MRI acquisition information, are available as a tab-separated values (.tsv) file accompanying the collection. All data can be accessed via the TCIA repository under the UTSW-Glioma collection.

## Code availability

Publicly available FeTS platform^29^ was used to process our data and generate automated tumor segmentations. The platform is accessible at https://github.com/FETS-AI/Front-End.
